# Is Yellow Fever Contagious?

**Published:** 1882-09

**Authors:** H. Rosencrans

**Affiliations:** Elgin, Ill.


### ARTICLE XI.

ELGIN, ILL., AUGUST 4, 1882.

EDITORS CHICAGO MEDICAL JOURNAL AND EXAMINER:

Is yellow fever contagious? My experience, extending through four epidemics of yellow fever, leads me to the belief that it is a non-contagious disease. In 1853 I had the sickness, and practiced through that epidemic in Lavaca, Texas. Out of seven physicians there, five died with the disease. Many ran away from the place, as is the case in all epidemics of like character. I attended a number of the refugees who only went out six miles, and camped on a bayou where there was a little settlement. No one took the disease in the settlement, notwithstanding they helped nurse the sick. The next season, 1854, the fever again appeared. Again in 1863 and 1867, the two towns of Lavaca and Indianola suffered greatly with the sickness. During every one of these epidemics many fled. I never knew of a case of fever resulting from exposure to it, nor from infected clothing *fomites*. I think the only method of finding out if the sickness be contagious is for some one to take it from some such exposure, but with all my experience, I never knew such a case. I have witnessed sporadic cases of it in New Orleans and in other places up to 1878; treated it on shipboard; held post-mortem examinations, and had friends and others present. I have had bedclothing washed and preserved on which the sick and dying had lain, the bedding besmeared with black vomit; never a case occurring from any such exposure within my knowledge. I do not think there are any well authenticated cases of the kind.

I do not believe that in an infected place, where the disease is prevailing as an epidemic, it can be proven that one person takes it from another. I have always observed that in the beginning of this sickness in a place, as an epidemic, the sickness appears suddenly and simultaneously in different and remote spots, showing the disease to have an atmospheric origin. My experience also teaches me that its cause is a domestic one, not depending on any exotic principle. I have had the singular fortune to meet with and treat the first cases in no less than three epidemics, and under such circumstances as to preclude the possibility of any foreign origin. H. ROSENCRANS, M.D.

---

### NATIONAL CONFERENCE OF CHARITIES AND CORRECTIONS.

The ninth annual session of the above organization was held in Madison, Wisconsin, commencing August 7, 1882, and continued five days. The attendance was large, and a wide range of topics relating to charitable and penal institutions were discussed. We are glad to notice that some, at least, out of the many who are devoting attention to these important matters, are beginning to expose the folly of investing so many millions of dollars in imposing public edifices for the protection or correction of classes who would be far better served, and the interests of the people also better protected, by less magnificence in building, and more attention to the moral and physical condition of those placed under public charge. D.

